# Supplementary material for: Microwave-Assisted Synthesis, Biological Activity Evaluation, Molecular Docking, and ADMET Studies of Some Novel Pyrrolo [2,3-b] Pyrrole Derivatives
Source: Molecules. 2022 Mar 23;27(7):2061. doi: 10.3390/molecules27072061 (PMC9000376; doi:10.3390/molecules27072061)
Supplement: Supplementary file 1 [file molecules-27-02061-s001.zip › molecules-1612668-supplementary.pdf]

M-4
proton_su DMSO {C:\nmr-data} Student 9

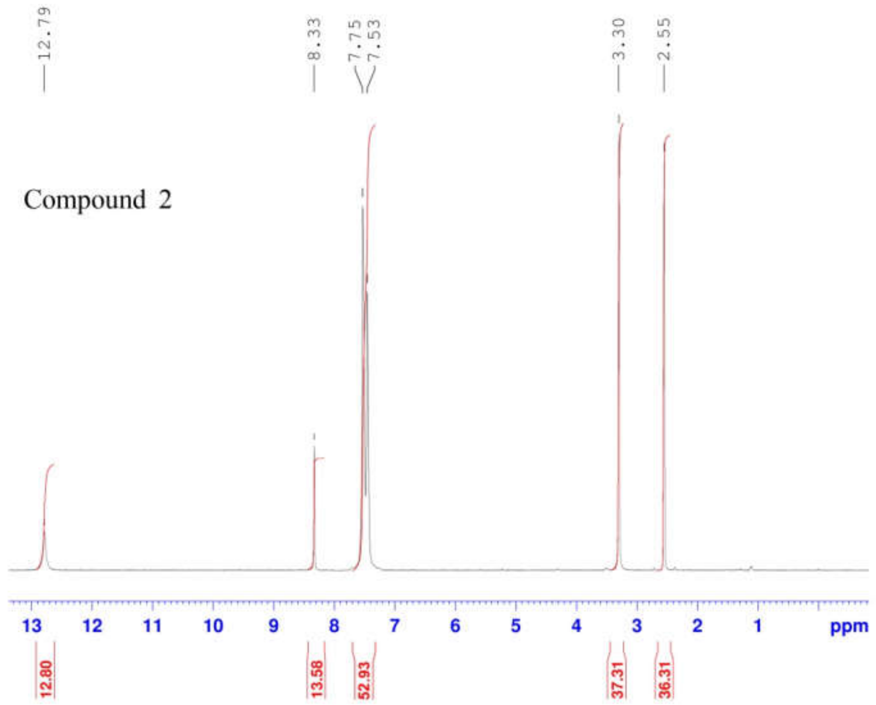


```
Current Data Parameters
NAME              Feb28-2017
EXPNO                    290
PROCNO                     1

F2 - Acquisition Parameters
Date_               20170228
Time                   16.17
INSTRUM                spect
PROBHD     5 mm PABBO BB/
PULPROG                 zg30
TD                     65536
SOLVENT                 DMSO
NS                        50
DS                         2
SWH                 8012.820 Hz
FIDRES              0.122266 Hz
AQ                 4.0894465 sec
RG                    199.04
DW                    62.400 usec
DE                      6.50 usec
TE                     313.1 K
D1                1.00000000 sec
TD0                        1

======== CHANNEL f1 ========
SFO1             400.1324710 MHz
NUC1                      1H
P1                     12.00 usec
PLW1             22.00000000 W

F2 - Processing parameters
SI                     65536
SF               400.1299827 MHz
WDW                       EM
SSB          0
LB                      0.30 Hz
GB           0
PC                      1.00
```

MP-1
c13_su DMSO {C:\nmr-data} Student 13

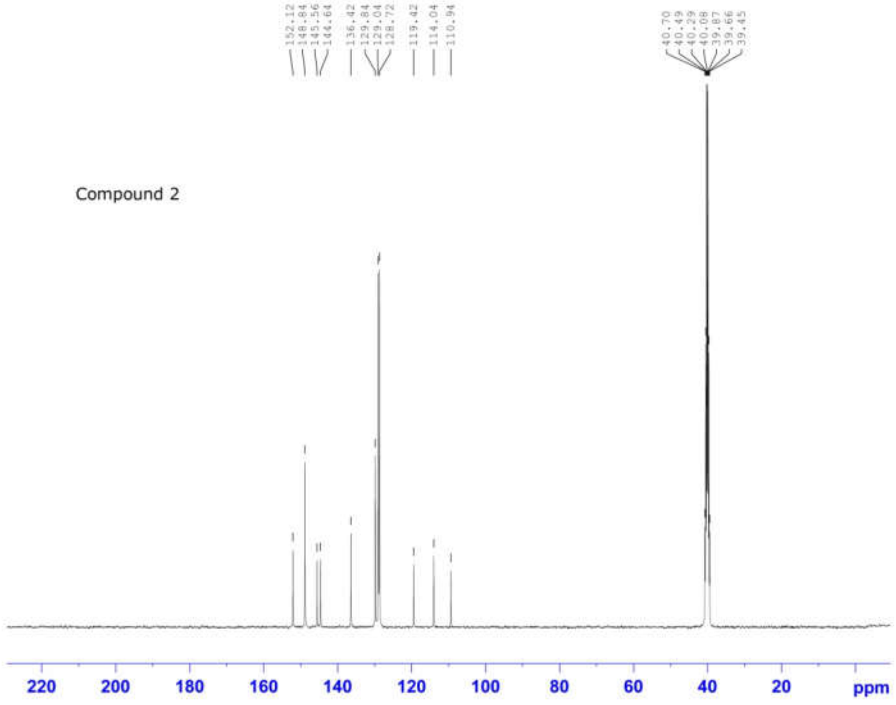


```
Current Data Parameters
NAME              Jan17-2018
EXPNO                    350
PROCNO                     1

F2 - Acquisition Parameters
Date_               20180118
Time                    0.15
INSTRUM                spect
PROBHD     5 mm PABBO BB/
PULPROG               zgpg30
TD                     65536
SOLVENT                 DMSO
NS                      1200
DS                         4
SWH                24038.461 Hz
FIDRES              0.366798 Hz
AQ                 1.3631488 sec
RG                    100.43
DW                    20.800 usec
DE                      6.50 usec
TE                     308.1 K
D1                2.00000000 sec
D11               0.03000000 sec
TD0                        1

======== CHANNEL f1 ========
SFO1             100.6238364 MHz
NUC1                     13C
P1                      9.50 usec
PLW1             56.00000000 W

======== CHANNEL f2 ========
SFO2             400.1316005 MHz
NUC2                      1H
CPDPRG[2             waltz16
PCPD2                  90.00 usec
PLW2             22.00000000 W
PLW12             0.41091001 W
PLW13             0.33284000 W

F2 - Processing parameters
SI                     32768
SF               100.6127690 MHz
WDW                       EM
SSB          0
LB                      6.00 Hz
GB           0
PC                      1.40
```

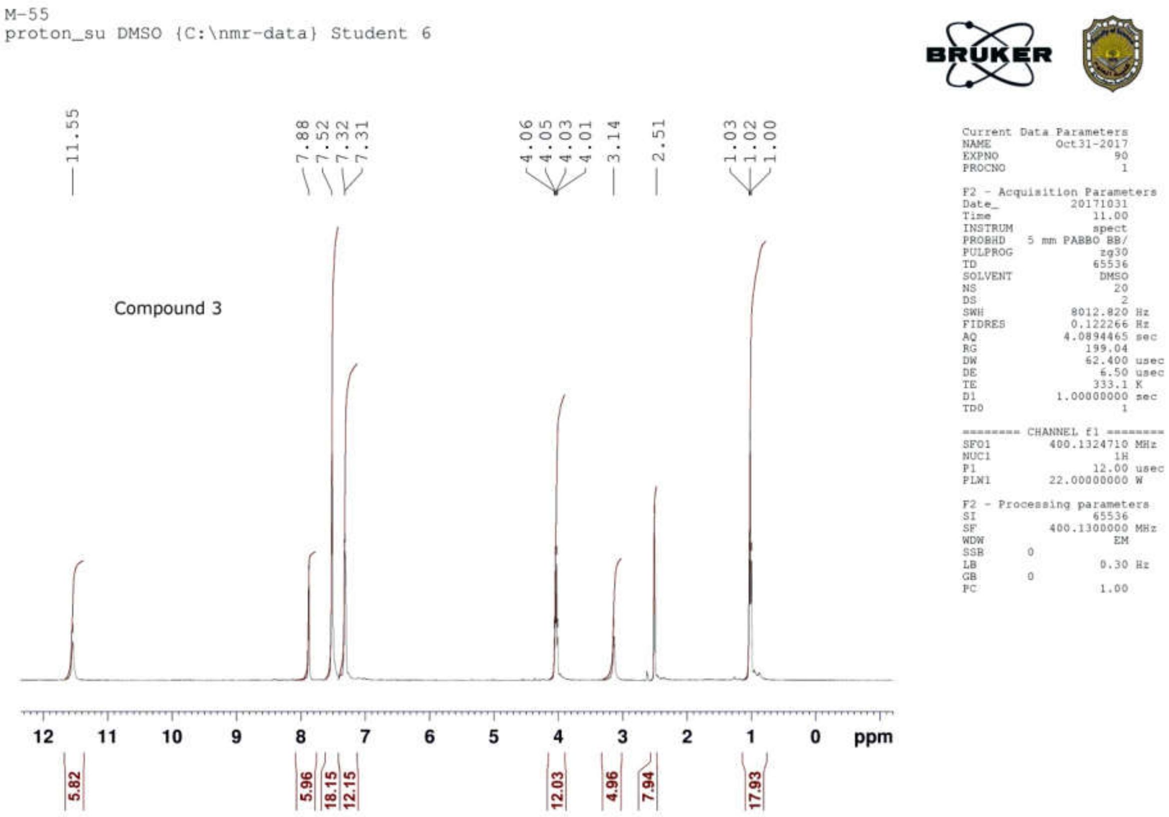
M-55
proton_su DMSO {C:\nmr-data} Student 6
BRUKER
11.55
7.88
7.52
7.32
7.31
4.06
4.05
4.03
4.01
3.14
2.51
1.03
1.02
1.00
Compound 3
12 11 10 9 8 7 6 5 4 3 2 1 0 ppm
5.82
5.96
18.15
12.15
12.03
4.96
7.94
17.93
Current Data Parameters
NAME Oct31-2017
EXPNO 90
PROCNO 1
F2 - Acquisition Parameters
Date_ 20171031
Time 11.00
INSTRUM spect
PROBHD 5 mm PABBO BB/
PULPROG zg30
TD 65536
SOLVENT DMSO
NS 20
DS 2
SWH 8012.820 Hz
FIDRES 0.122266 Hz
AQ 4.0894465 sec
RG 199.04
DW 62.400 usec
DE 6.50 usec
TE 333.1 K
D1 1.00000000 sec
TD0 1
======== CHANNEL f1 ========
SFO1 400.1324710 MHz
NUC1 1H
P1 12.00 usec
PLW1 22.00000000 W
F2 - Processing parameters
SI 65536
SF 400.1300000 MHz
WDW EM
SSB 0
LB 0.30 Hz
GB 0
PC 1.00

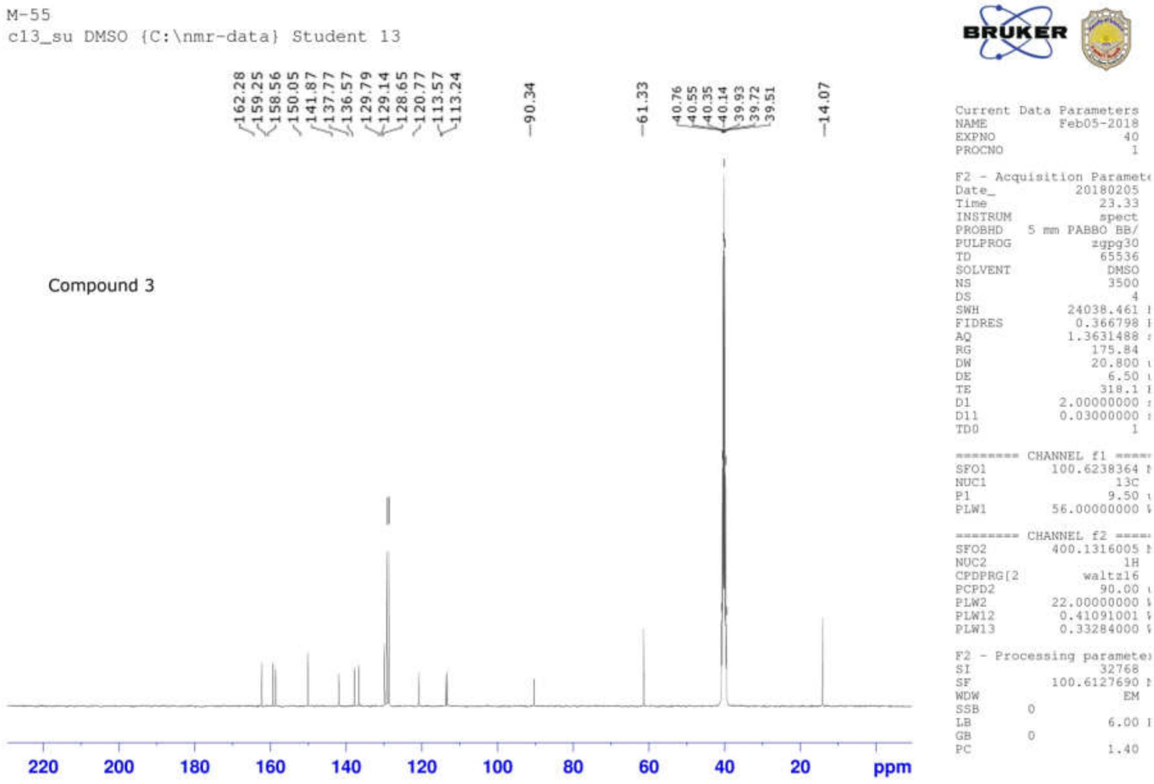
M-55
c13_su DMSO {C:\nmr-data} Student 13
BRUKER
162.28
159.25
158.56
150.05
141.87
137.77
136.57
129.79
129.14
128.65
120.77
113.57
113.24
90.34
61.33
40.76
40.55
40.35
40.14
39.93
39.72
39.51
14.07
Compound 3
220 200 180 160 140 120 100 80 60 40 20 ppm
Current Data Parameters
NAME Feb05-2018
EXPNO 40
PROCNO 1
F2 - Acquisition Paramet
Date_ 20180205
Time 23.33
INSTRUM spect
PROBHD 5 mm PABBO BB/
PULPROG zgpg30
TD 65536
SOLVENT DMSO
NS 3500
DS 4
SWH 24038.461
FIDRES 0.366798
AQ 1.3631488
RG 175.84
DW 20.800
DE 6.50
TE 318.1
D1 2.00000000
D11 0.03000000
TD0 1
======== CHANNEL f1 ====
SFO1 100.6238364
NUC1 13C
P1 9.50
PLW1 56.00000000
======== CHANNEL f2 ====
SFO2 400.1316005
NUC2 1H
CPDPRG[2 waltz16
PCPD2 90.00
PLW2 22.00000000
PLW12 0.41091001
PLW13 0.33284000
F2 - Processing paramete
SI 32768
SF 100.6127690
WDW EM
SSB 0
LB 6.00
GB 0
PC 1.40

MP-2
proton_su DMSO {C:\nmr-data} Student 17

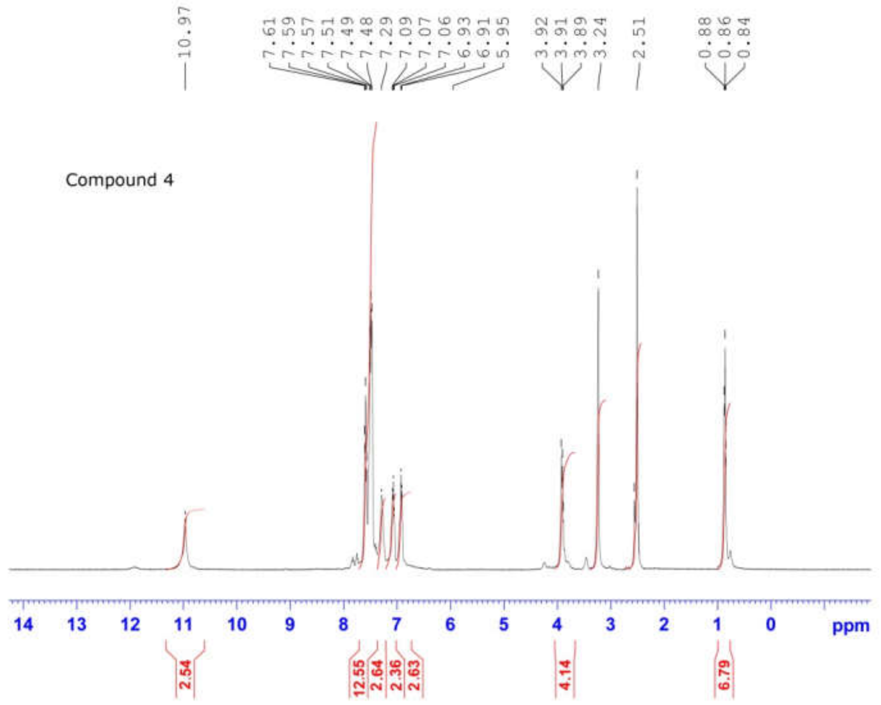

Current Data Parameters
NAME Jul26-2017
EXPNO 150
PROCNO 1

F2 - Acquisition Parameters
Date_ 20170726
Time 12.39
INSTRUM spect
PROBHD 5 mm PABBO BB/
PULPROG zg30
TD 65536
SOLVENT DMSO
NS 50
DS 2
SWH 8012.820 Hz
FIDRES 0.122266 Hz
AQ 4.0894465 sec
RG 199.04
DW 62.400 usec
DE 6.50 usec
TE 318.2 K
D1 1.00000000 sec
TD0 1

======== CHANNEL f1 ========
SFO1 400.1324710 MHz
NUC1 1H
P1 12.00 usec
PLW1 22.00000000 W

F2 - Processing parameters
SI 65536
SF 400.1300000 MHz
WDW EM
SSB 0
LB 0.30 Hz
GB 0
PC 1.00

MP-2
c13_su DMSO {C:\nmr-data} Student 13

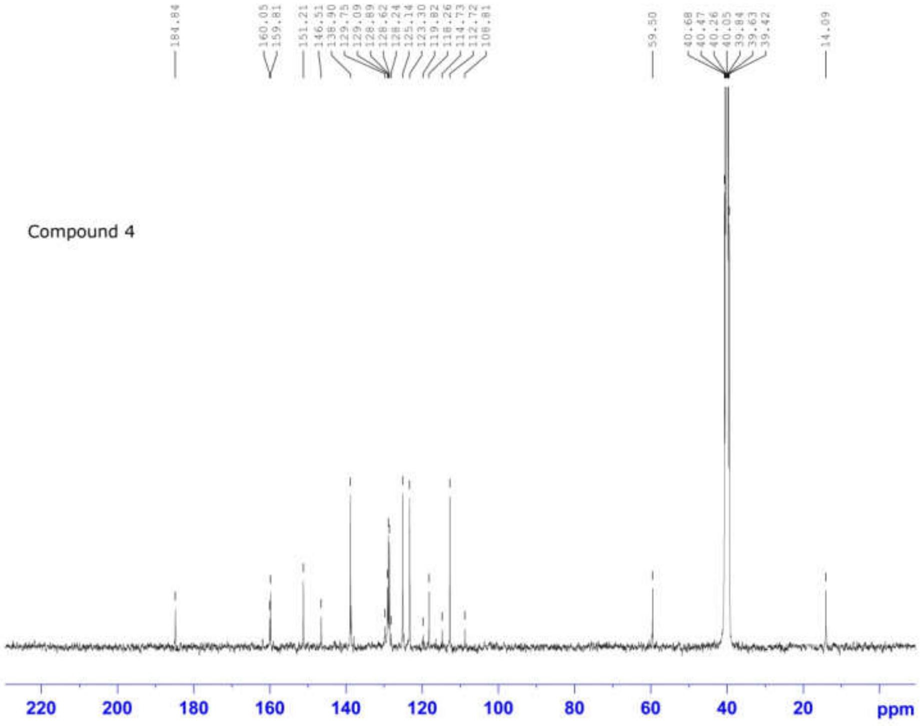

Current Data Parameters
NAME Oct02-2017
EXPNO 12
PROCNO 1

F2 - Acquisition Parameters
Date_ 20171002
Time 16.40
INSTRUM spect
PROBHD 5 mm PABBO BB/
PULPROG zgpg30
TD 65536
SOLVENT DMSO
NS 2500
DS 4
SWH 24038.461 Hz
FIDRES 0.366798 Hz
AQ 1.3631488 sec
RG 158.76
DW 20.800 usec
DE 6.50 usec
TE 318.2 K
D1 2.00000000 sec
D11 0.03000000 sec
TD0 1

======== CHANNEL f1 ========
SFO1 100.6238364 MHz
NUC1 13C
P1 9.50 usec
PLW1 56.00000000 W

======== CHANNEL f2 ========
SFO2 400.1316005 MHz
NUC2 1H
CPDPRG[2 waltz16
PCPD2 90.00 usec
PLW2 22.00000000 W
PLW12 0.41091001 W
PLW13 0.33284000 W

F2 - Processing parameters
SI 32768
SF 100.6127690 MHz
WDW EM
SSB 0
LB 6.00 Hz
GB 0
PC 1.40

MP-6
proton_su DMSO {C:\nmr-data} Student 22

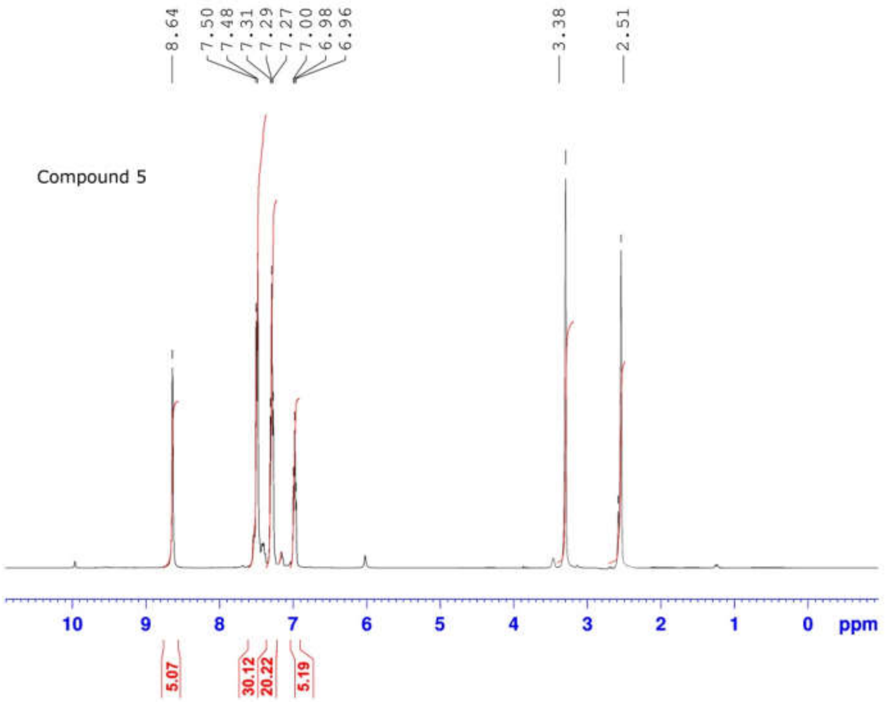


```
Current Data Parameters
NAME          Dec17-2017
EXPNO                 20
PROCNO                 1

F2 - Acquisition Parameters
Date_           20171217
Time               12.57
INSTRUM            spect
PROBHD   5 mm PABBO BB/
PULPROG             zg30
TD                 65536
SOLVENT             DMSO
NS                   100
DS                     2
SWH           8012.820 Hz
FIDRES        0.122266 Hz
AQ           4.0894465 sec
RG                 49.89
DW                62.400 usec
DE                  6.50 usec
TE                 308.2 K
D1          1.00000000 sec
TD0                    1

======== CHANNEL f1 ========
SFO1        400.1324710 MHz
NUC1                  1H
P1                 12.00 usec
PLW1         22.00000000 W

F2 - Processing parameters
SI                 65536
SF          400.1300000 MHz
WDW                   EM
SSB      0
LB                  0.30 Hz
GB       0
PC                  1.00
```

MP-6
c13_su DMSO {C:\nmr-data} Student 10

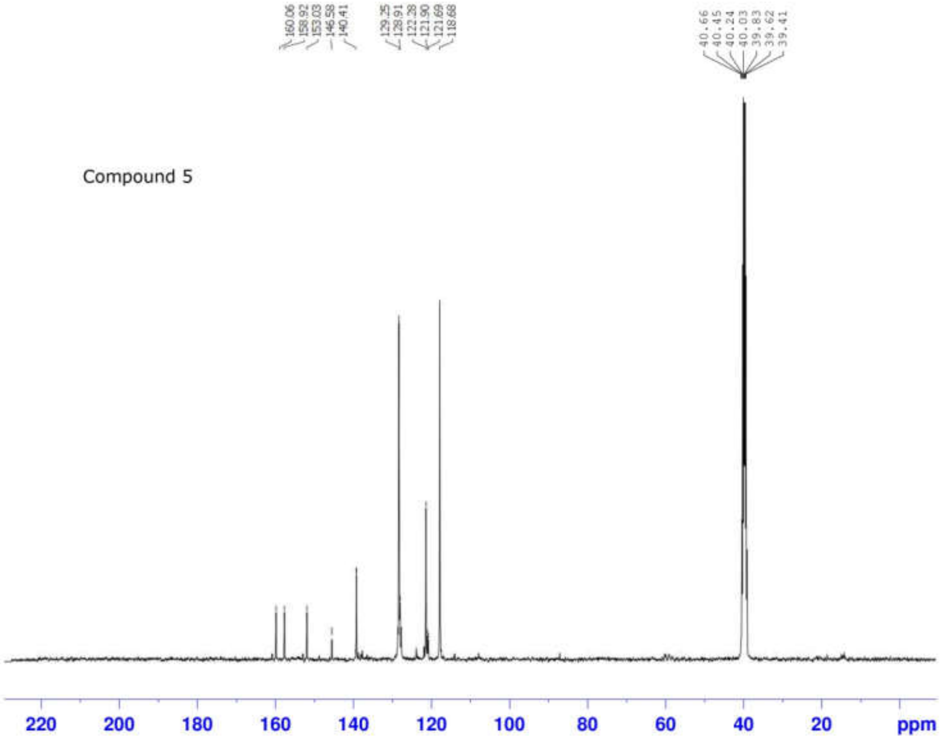


```
Current Data Parameters
NAME          Dec21-2017
EXPNO                 90
PROCNO                 1

F2 - Acquisition Paramet
Date_           20171221
Time               12.44
INSTRUM            spect
PROBHD   5 mm PABBO BB/
PULPROG           zgpg30
TD                 65536
SOLVENT             DMSO
NS                  1100
DS                     4
SWH            24038.461
FIDRES          0.366798
AQ             1.3631488
RG                158.76
DW                20.800
DE                  6.50
TE                 303.1
D1            2.00000000
D11           0.03000000
TD0                    1

======== CHANNEL f1 ====
SFO1        100.6238364
NUC1                 13C
P1                  9.50
PLW1         56.00000000

======== CHANNEL f2 ====
SFO2        400.1316005
NUC2                  1H
CPDPRG[2         waltz16
PCPD2              90.00
PLW2         22.00000000
PLW12         0.41091001
PLW13         0.33284000

F2 - Processing paramete
SI                 32768
SF          100.6127690
WDW                   EM
SSB      0
LB                  6.00
GB       0
PC                  1.40
```

M-54
proton_su DMSO {C:\nmr-data} Student 6

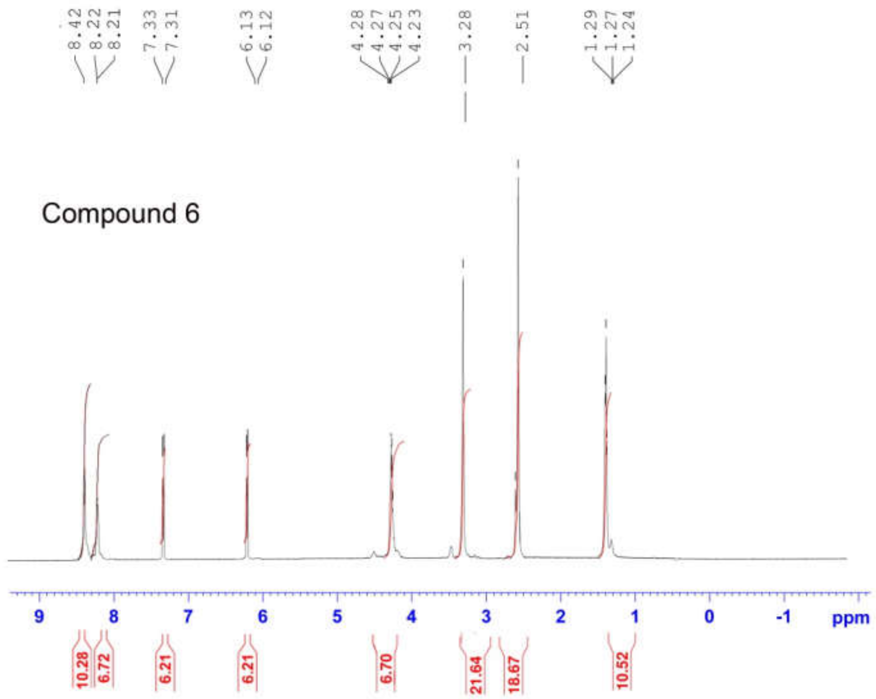


```
Current Data Parameters
NAME           Sep20-2017
EXPNO                  40
PROCNO                  1

F2 - Acquisition Parameters
Date_            20170920
Time                10.04
INSTRUM             spect
PROBHD   5 mm PABBO BB/
PULPROG              zg30
TD                  65536
SOLVENT              DMSO
NS                     20
DS                      2
SWH              8012.820 Hz
FIDRES           0.122266 Hz
AQ              4.0894465 sec
RG                 199.04
DW                 62.400 usec
DE                   6.50 usec
TE                  308.1 K
D1             1.00000000 sec
TD0                     1

======== CHANNEL f1 ========
SFO1          400.1324710 MHz
NUC1                   1H
P1                  12.00 usec
PLW1          22.00000000 W

F2 - Processing parameters
SI                  65536
SF            400.1300000 MHz
WDW                    EM
SSB      0
LB                   0.30 Hz
GB       0
PC                   1.00
```

M-54
c13_su DMSO {C:\nmr-data} Student 16

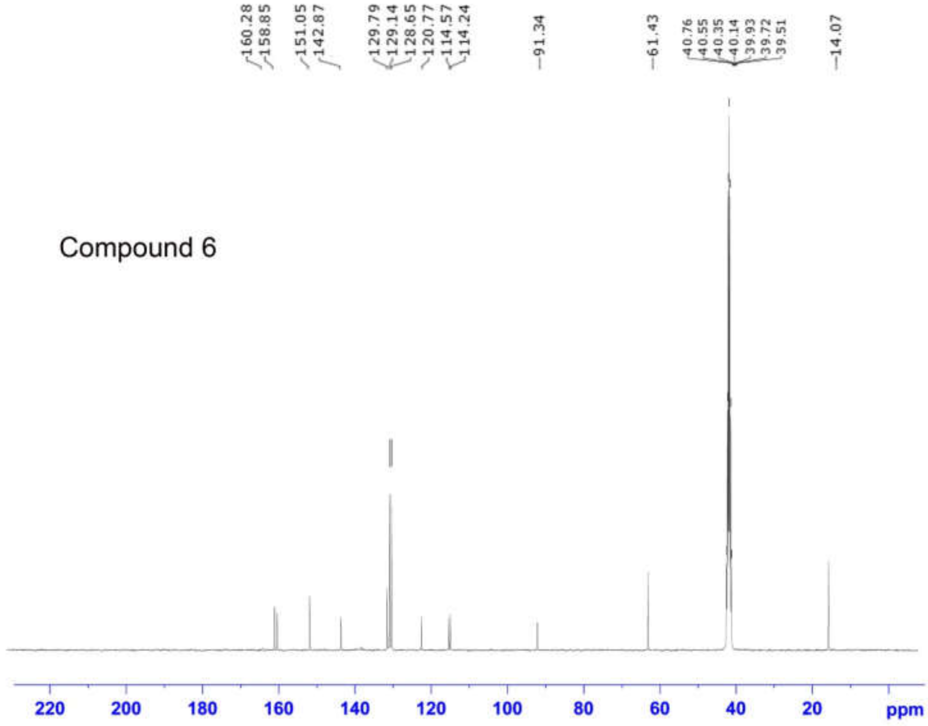


```
Current Data Parameters
NAME           Oct02-2017
EXPNO                  40
PROCNO                  1

F2 - Acquisition Parameters
Date_            20171002
Time                23.30
INSTRUM             spect
PROBHD   5 mm PABBO BB/
PULPROG            zgpg30
TD                  65536
SOLVENT              DMSO
NS                   2400
DS                      4
SWH             24038.461 Hz
FIDRES           0.366798 Hz
AQ              1.3631488 sec
RG                 199.04
DW                 20.800 usec
DE                   6.50 usec
TE                  318.2 K
D1             2.00000000 sec
D11            0.03000000 sec
TD0                     1

======== CHANNEL f1 ========
SFO1          100.6238364 MHz
NUC1                  13C
P1                   9.50 usec
PLW1          56.00000000 W

======== CHANNEL f2 ========
SFO2          400.1316005 MHz
NUC2                   1H
CPDPRG[2          waltz16
PCPD2               90.00 usec
PLW2          22.00000000 W
PLW12          0.41091001 W
PLW13          0.33284000 W

F2 - Processing parameters
SI                  32768
SF            100.6127690 MHz
WDW                    EM
SSB      0
LB                   6.00 Hz
GB       0
PC                   1.40
```

mp-4
proton_su DMSO {C:\nmr-data} Student 3

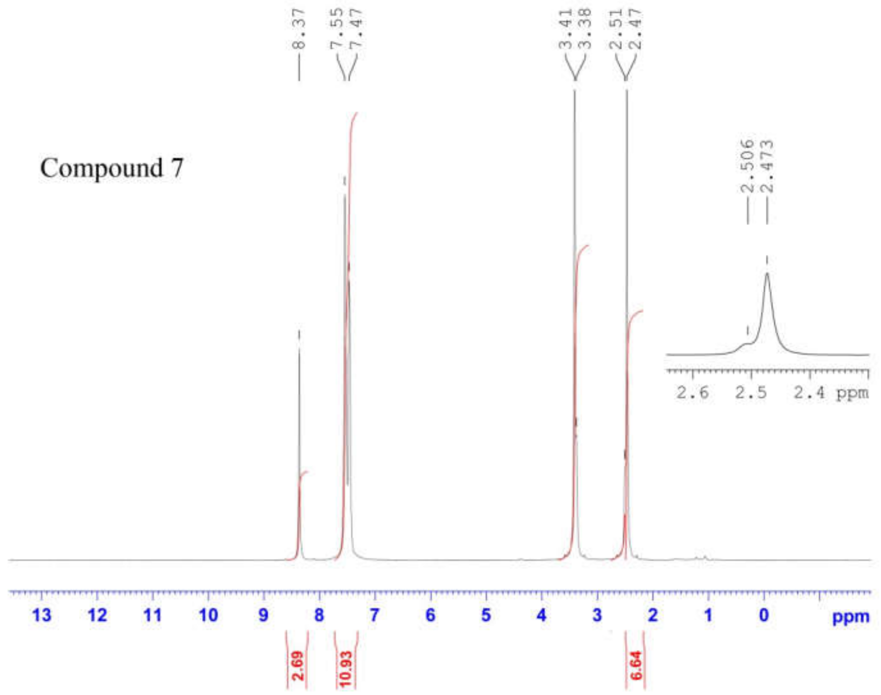

Current Data Parameters
NAME Dec20-2017
EXPNO 10
PROCNO 1

F2 - Acquisition Parameters
Date_ 20171220
Time 11.47
INSTRUM spect
PROBHD 5 mm PABBO BB/
PULPROG zg30
TD 65536
SOLVENT DMSO
NS 20
DS 2
SWH 8012.820 Hz
FIDRES 0.122266 Hz
AQ 4.0894465 sec
RG 68.22
DW 62.400 usec
DE 6.50 usec
TE 293.5 K
D1 1.00000000 sec
TD0 1

======== CHANNEL f1 ========
SFO1 400.1324710 MHz
NUC1 1H
P1 12.00 usec
PLW1 22.00000000 W

F2 - Processing parameters
SI 65536
SF 400.1300000 MHz
WDW EM
SSB 0
LB 0.30 Hz
GB 0
PC 1.00

MP-4
c13_su DMSO {C:\nmr-data} Student 13

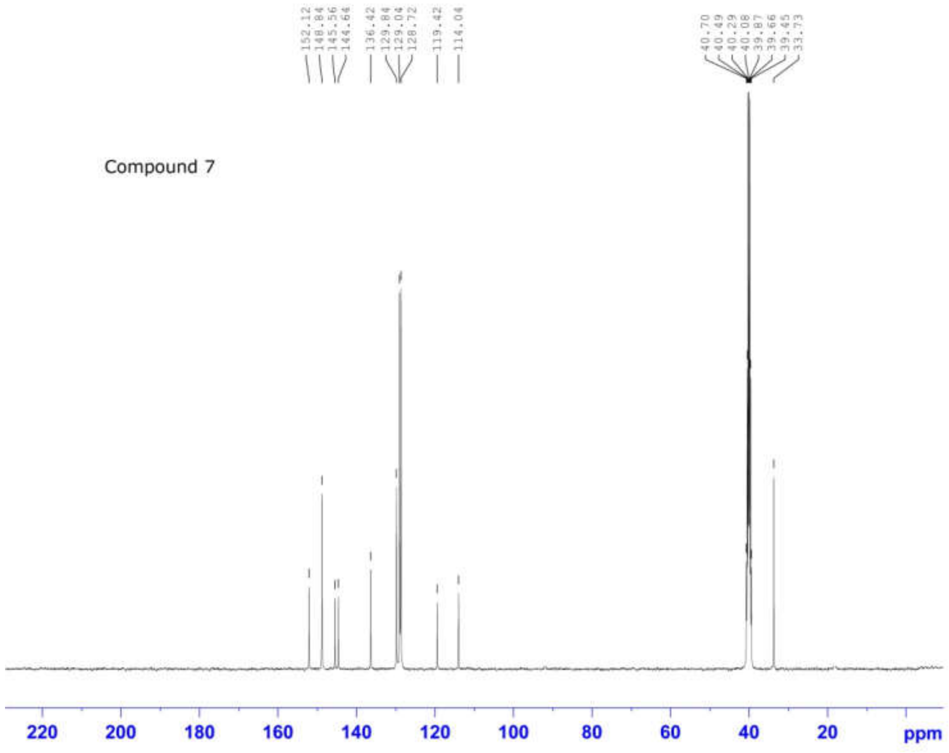

Current Data Parameters
NAME Jan17-2018
EXPNO 350
PROCNO 1

F2 - Acquisition Parameters
Date_ 20180118
Time 0.15
INSTRUM spect
PROBHD 5 mm PABBO BB/
PULPROG zgpg30
TD 65536
SOLVENT DMSO
NS 1200
DS 4
SWH 24038.461 Hz
FIDRES 0.366798 Hz
AQ 1.3631488 sec
RG 100.43
DW 20.800 usec
DE 6.50 usec
TE 308.1 K
D1 2.00000000 sec
D11 0.03000000 sec
TD0 1

======== CHANNEL f1 ========
SFO1 100.6238364 MHz
NUC1 13C
P1 9.50 usec
PLW1 56.00000000 W

======== CHANNEL f2 ========
SFO2 400.1316005 MHz
NUC2 1H
CPDPRG[2 waltz16
PCPD2 90.00 usec
PLW2 22.00000000 W
PLW12 0.41091001 W
PLW13 0.33284000 W

F2 - Processing parameters
SI 32768
SF 100.6127690 MHz
WDW EM
SSB 0
LB 6.00 Hz
GB 0
PC 1.40
